# Exercise prescription to improve inhibitory control in children and adolescents with ADHD: a network meta-analysis

**DOI:** 10.3389/fpsyt.2025.1601765

**Published:** 2025-06-30

**Authors:** Ke Zhao, Yongxiao Li, Penglong Wang, Ping Zhang

**Affiliations:** ^1^ Postgraduate School, University of Harbin Sport, Harbin, China; ^2^ College of Physical Education, Zhangjiakou University, Zhangjiakou, Hebei, China; ^3^ The Sports Academy, Jiangsu Ocean University, Lianyungang, Jiangsu, China

**Keywords:** exercise prescription, inhibitory control, ADHD, children and adolescents, meta-analysis

## Abstract

**Objective:**

To investigate the effects of different combinations of exercise prescription variables (type, duration, frequency, period, intensity) on inhibitory control in children and adolescents with ADHD, and to provide a basis for the development of exercise prescriptions for intervening inhibitory control in children and adolescents with ADHD.

**Methods:**

Data sources were searched up to February 1, 2025, including Scopus, PubMed, Web of Science, Embase, and Cochrane. The Cochrane Risk of Bias Assessment Tool was utilized for methodological quality assessment. Stata 17.0 software was used for net meta-analysis to compare the interventions with each other, using standardized mean difference (SMD) and 95% CI as the effect indicators. The effect differences between the interventions were compared using standardized mean difference (SMD) and 95% CI as effect indicators, and the effects of exercise prescription variables were ranked using Surface Under the Cumulative Ranking Area (SUCRA).

**Results:**

Twenty papers with 1450 participants aged 7–18 years were finally included. Net Meta-analysis showed that the intervention duration of 70 minutes (SMD=2.15, 95% CI (1.02,3.28)) was significantly better than the control group. The intervention effect was significantly better for 2 times per week (SMD=1.27, 95% CI: (0.65,1.90)) than for the control group, and the intervention effect for at least 20 weeks (SMD=1.37, 95% CI (0.32,2.41)) was significantly better than the intervention effect for 12 weeks. moderate to high intensity (SMD = -0.14, 95% CI (-0.68, -0.40)) was significantly lower than moderate intensity (all P values < 0.05). The ranking of the SUCRA results showed that the intervention effect of Taekwondo (SUCRA=87.1), 70 minutes/repetition (SUCRA=99.0), twice per week (SUCRA=97.8), and continued for at least 20 weeks (SUCRA=92.5) and moderate intervention intensity (SUCRA=98.9) may have the best effect on inhibitory control effects in children and adolescents with ADHD.

**Conclusion:**

This study found that for the improvement of inhibitory control in children and adolescents with ADHD, a moderate-intensity taekwondo exercise intervention model of 70 minutes twice a week for at least 20 weeks can achieve more satisfactory results, which provides a program choice with reference value for relevant clinical intervention practice.

**Systematic review registration:**

https://www.crd.york.ac.uk/PROSPERO/, identifier CRD420251019338.

## 1 Introduction

Attention deficit hyperactivity disorder (ADHD) is the most common neurodevelopmental disorder among children and adolescents, with a global prevalence of ADHD of approximately 5-7% (1). The core symptoms of ADHD include inattentiveness, hyperactivity, and impulsive behaviors, which have a serious impact on children and adolescents’ academic, social, and family lives (2). One of the major functional deficits in ADHD is inhibitory control deficit, which is manifested by poor impulse control and difficulties in behavioral regulation (3, 4). Although medication is currently the mainstay of treatment for ADHD, drugs may cause side effects such as loss of appetite, insomnia, and anxiety, which has prompted researchers to look for other non-pharmacological interventions to minimize the side effects of drugs (5).

In recent years, physical activity as a non-pharmacological intervention has gradually gained widespread attention from researchers, and relevant studies have shown that physical activity can improve nerve conduction, regulate neurotransmitter levels, and enhance inhibitory control in children and adolescents with ADHD, thereby alleviating the symptoms of ADHD (6, 7). On this basis, exercise can promote the optimization of neural activity patterns by improving brain activity, especially adaptive changes in the prefrontal cortex, which has a significant effect on improving attention, impulse control, and behavioral inhibition in children and adolescents with ADHD (8). Guidelines organized by the Canadian Paediatric Society (CPS) also recommend physical activity as an effective means of ADHD treatment, emphasizing the ability of exercise to improve behavioral performance in children and adolescents with ADHD (9, 10). Hua et al. have proposed in the literature to investigate the effects of different exercise prescription variables on improving inhibitory control in patients with ADHD through a neurobiological mechanism (10). Inhibitory control effects in ADHD patients (11). While exercise prescription guidelines are based on the FITT principle (Frequency, Intensity, Time, Type), each element is interrelated and interacts with each other, and a rational design can significantly enhance the effectiveness of the intervention (12).

Although meta-studies have been conducted to show that exercise interventions have a positive effect on inhibitory control in participants with ADHD (13, 14), as Yang et al. found that cognitive-motor training was the most effective for inhibitory control in their meta-analysis (13), and Zhu et al. mentioned that open-skill activities were the most effective in improving inhibitory control in children with ADHD in their meta-analysis (15). However, systematic comparisons of the effects of factors such as exercise type, intensity, and frequency on intervention effects are still scarce. The advantage of reticulated Meta-analysis is that it can indirectly compare the effects of various interventions by using the effects of multiple interventions as a mediator, which can overcome the limitation that traditional Meta-analysis can only deal with the direct comparison of two interventions even if there is a lack of direct comparative evidence, thus improving the precision of the analysis (4). However, there is currently no review that explores the effect of the FITT variable on the inhibitory control of children with ADHD, therefore, this study aimed to provide recommendations for exercise programs to alleviate inhibitory control in children and adolescents with ADHD by conducting a reticulated Meta-analysis of randomized controlled trials of inhibitory control in children and adolescents with ADHD.

## 2 Methods

This systematic evaluation was conducted following the requirements of the Priority Reporting Entries for Systematic Reviews and Meta-Analyses: a PRISMA Statement and was registered on the prospective registry platform PROSPERO under the registration number CRD420251019338 (16). Also, and results were reported according to the network meta-analysis extended reporting guidelines (PRISMA-NMA) (17).

### 2.1 Literature search strategy

Five databases were searched: PubMed, Web of Science, Embase, Scopus, and Cochrane, with the search date ending on February 20, 2025, from the start date of each database. The following combinations of search terms were used: ①exercise, Strength Training, physical exercise, physical activity, sports, fitness, Functional Training, Exercise Therapy; ② ADHD children, ADHD children, elementary school students, adolescents, young adults, school-aged children, school-aged people; ③ executive functioning, working memory, inhibitory control, cognitive flexibility, goal-directed behavior, task switching, short-term memory, retentive retraining, impulse control, response inhibition, interference inhibition, transitive stereotyping, multitasking, RCT, experiment, trial through Boolean logical operators “AND” was used to connect the 3 groups of words, in addition to the inclusion of studies by tracking the literature in published systematic evaluations and meta-analyses to ensure the comprehensiveness of the retrieved literature. Taking Web of Science as an example, the search formula is: TS=(exercise OR “Strength Training” OR “physical exercise” OR “physical activity” OR sports OR fitness OR “Functional Training” OR ‘Exercise Therapy’) AND TS=(“ADHD children” OR ‘primary school students’ OR adolescents OR juvenile OR ‘school children’ OR “school-age population”) AND TS=(“executive functions” OR ‘working memory’ OR ‘inhibitory control’ OR ‘cognitive flexibility’ OR ‘goal - directed behavior’ OR “ task - switching” OR ‘short - term memory’ OR ‘maintenance rehearsal’ OR ‘impulse control’ OR ‘response inhibition’ OR ‘interference inhibition’ OR “ set - shifting” OR ‘multitasking’ OR RCT OR experiment OR trial). Details of the search strategy are given in **Table 1**.

**Table 1:** Complete search strategy for databases.

Database	Search Strategy
Pubmed	((exercise[MeSH Terms] OR "Strength Training"[MeSH Terms] OR "physical exercise"[MeSH Terms] OR "physical activity"[MeSH Terms] OR sports[MeSH Terms] OR fitness[MeSH Terms] OR "Functional Training"[MeSH Terms] OR "Exercise Therapy"[MeSH Terms]) AND ("ADHD"[MeSH Terms] AND children[MeSH Terms] OR "primary school students"[MeSH Terms] OR adolescents[MeSH Terms] OR juvenile[MeSH Terms] OR "school children"[MeSH Terms] OR "school-age population"[MeSH Terms]) AND ("Executive Function"[MeSH Terms] OR "Working Memory"[MeSH Terms] OR "Inhibitory Control"[MeSH Terms] OR "Cognitive Flexibility"[MeSH Terms] OR "Goal-Directed Behavior"[MeSH Terms] OR "Task Switching"[MeSH Terms] OR "Short-Term Memory"[MeSH Terms] OR "Maintenance Rehearsal"[MeSH Terms] OR "Impulse Control"[MeSH Terms] OR "Response Inhibition"[MeSH Terms] OR "Interference Inhibition"[MeSH Terms] OR "Set Shifting"[MeSH Terms] OR "Multitasking"[MeSH Terms] OR "RCT"[MeSH Terms] OR "experiment"[MeSH Terms] OR "trial"[MeSH Terms]))
Web of science	TS=(exercise OR "Strength Training" OR "physical exercise" OR "physical activity" OR sports OR fitness OR "Functional Training" OR "Exercise Therapy") AND TS=("ADHD children" OR "primary school students" OR adolescents OR juvenile OR "school children" OR "school-age population") AND TS=("executive functions" OR "working memory" OR "inhibitory control" OR "cognitive flexibility" OR "goal-directed behavior" OR "task-switching" OR "short-term memory" OR "maintenance rehearsal" OR "impulse control" OR "response inhibition" OR "interference inhibition" OR "set-shifting" OR "multitasking" OR RCT OR experiment OR trial)
Embase	1. (exercise OR "Strength Training" OR "physical exercise" OR "physical activity" OR sports OR fitness OR "Functional Training" OR "Exercise Therapy") [emtree]/exp 2. ("ADHD children" OR "primary school students" OR adolescents OR juvenile OR "school children" OR "school-age population") [emtree]/exp 3. ("executive functions" OR "working memory" OR "inhibitory control" OR "cognitive flexibility" OR "goal-directed behavior" OR "task-switching" OR "short-term memory" OR "maintenance rehearsal" OR "impulse control" OR "response inhibition" OR "interference inhibition" OR "set-shifting" OR "multitasking" OR RCT OR experiment OR trial) [emtree]/exp 4. 1 AND 2 AND 3
Scopus	1. TITLE-ABS-KEY(exercise OR "Strength Training" OR "physical exercise" OR "physical activity" OR sports OR fitness OR "Functional Training" OR "Exercise Therapy") 2. TITLE-ABS-KEY("ADHD children" OR "primary school students" OR adolescents OR juvenile OR "school children" OR "school-age population") 3. TITLE-ABS-KEY("executive functions" OR "working memory" OR "inhibitory control" OR "cognitive flexibility" OR "goal-directed behavior" OR "task-switching" OR "short -term memory" OR "maintenance rehearsal" OR "impulse control" OR "response inhibition" OR "interference inhibition" OR "set-shifting" OR "multitasking" OR RCT OR experiment OR trial) 4. 1 AND 2 AND 3
Cochrane	1. (exercise OR "Strength Training" OR "physical exercise" OR "physical activity" OR sports OR fitness OR "Functional Training" OR "Exercise Therapy") [MeSH Terms] 2. ("ADHD children" OR "primary school students" OR adolescents OR juvenile OR "school children" OR "school-age population") [MeSH Terms] 3. ("executive functions" OR "working memory" OR "inhibitory control" OR "cognitive flexibility" OR "goal-directed behavior" OR "task-switching" OR "short-term memory" OR "maintenance rehearsal" OR "impulse control" OR "response inhibition" OR "interference inhibition" OR "set-shifting" OR "multitasking" OR RCT OR experiment OR trial) [MeSH Terms] 4. 1 AND 2 AND 3

### 2.2 Literature inclusion and exclusion criteria

Inclusion and exclusion criteria for the literature were developed according to the PICOS principle (18). The retrieved literature was de-duplicated by using EndNote 20 software (19). The titles and abstracts of the retrieved literature were initially screened by two independent evaluators using a double-blind approach based on the inclusion and exclusion criteria of the literature. Literature that might meet the inclusion criteria was downloaded in full text and re-screened to finalize the included literature. The inclusion criteria were as follows: (1) Population (Population): the study population was children and adolescents with attention deficit hyperactivity disorder (diagnosed according to the Diagnostic and Statistical Manual of Mental Disorders, fourth and fifth editions) under the age of 18 years. (2) Interventions: studies that included any form of exercise intervention, with no mandatory requirements for type, duration, intensity, and frequency of exercise, but with a minimum of 4 weeks of exercise intervention. (3) Control: The control group was treated in the usual way, including maintaining daily activities, health education, or routine care. (4) Outcomes: inhibitory control. (5) Study design: randomized controlled trial (RCT). Exclusion criteria were as follows: (1) studies focusing only on the physical fitness and motor skills of children and adolescents with ADHD; (2) studies using healthy children and adolescents as comparisons; (3) reviews, abstracts, letters, and commentaries that lacked a clear description of the study design; and (4) articles with incomplete data and where the required data could not be obtained by other means.

### 2.3 Data extraction

Literature screening and data extraction were done independently by two subject group members who are researchers with evidence-based methodology and have a long history of working with participants with ADHD (20). During the screening or data extraction process, the two researchers extracted independently, and in case of disagreement, they would discuss with the subject group members, negotiate together, and reach a consensus decision. During this process, the main focus was on extracting the following types of key information: the first author of the study, the date of publication of the article, the sample size of the participants, the type of intervention, the duration of the intervention, the frequency of the intervention, the period of the intervention, the intensity of the intervention, as well as the main outcome indicators used to assess the effectiveness of the intervention were entered into an Excel sheet and stored.

### 2.4 Risk of bias evaluation of included studies

In this study, the risk of bias assessment of all included studies was based on PROSPERO’s registration statement and was independently assessed using the Cochrane Risk of Bias Assessment Tool. The assessment framework covered the following seven aspects, including the validity of the randomization method, the implementation of blinding of trial participants and operators, the blinding status of the outcome assessors, the concealment of the allocation process, the completeness and accuracy of the outcome data, the presence of selective reporting of study results, and other potential bias factors. The quality risk of each study was also categorized into three categories: low risk, high risk, and uncertain risk. If any disagreement on risk of bias arises in the assessment, the assessors will reach a consensus through discussion. If consensus still cannot be reached, the corresponding author will make the final decision based on his/her judgment and taking into account the opinion of the majority of the assessors.

### 2.5 Statistical methods

This study was analyzed using Statа 17.0 software (21). Since the outcome indicators were continuous values and the evaluation tools and units used for individual studies were different, the standardized mean difference (SMD) was used as the effect size indicator to ensure consistency and comparability of the analyses and to accurately calculate the combined effect size. To further compare the efficacy of different interventions, we used the Surface Under the Cumulative Ranking Area (SUCRA) method to rank the interventions, with values ranging from 0 to 100; the higher the value, the better the efficacy of the effect, which further indicates the relative efficacy of the intervention in improving inhibitory control in participants with ADHD. SUCRA is a statistical method based on reticulated meta-analysis to quantify the relative efficacy of each intervention across all possible interventions. In reticulated meta-analysis, consistency reflects the similarity between the results of direct and indirect comparisons, and higher consistency indicates more reliable reticulated meta-analysis results. The global consistency test for the exercise intervention modality showed better global consistency for the type of exercise (P=0.235). Further consistency tests for closed rings showed that the lower limit of the inconsistency factor contained 0, indicating better consistency of the rings, and thus, the consistency model was used for the analysis. Single exercise time, exercise intervention frequency, exercise intervention period, and exercise intensity did not form a closed loop and therefore did not need to be tested for consistency.

### 2.6 Evidence certainty assessment

This study utilized the GRADE system assessment tool to evaluate the quality of evidence for all outcome indicators, and the results showed that there were 3 high-level evidence, 11 intermediate-level evidence, 5 low-level evidence, and 1 very low-level evidence (see **Table 2**). Among the downgrading factors, limitations were the main downgrading factor, 17 were downgraded because of limitations, most of the literature only mentioned randomization without describing the method of generating random numbers, most of the literature did not use blinding and allocation concealment, and only a few of the literature described the method of single-blind or double-blind and allocation concealment. , 

**Table 2:** Evaluation of the quality of evidence in the included literature.

Author & Year	Limitations	Inconsistency	Indirectness	Imprecision	Publication bias	Level of Evidence
Verret et al,2012 (22)	-1	0	0	0	0	Moderate
Ziereis et al,2015 (23)	-1	0	0	0	0	Moderate
Ji et al,2022 (24)	-1	0	0	0	0	Moderate
Kadri et al,2019 (25)	-1	0	0	0	0	Moderate
Memarmoghaddam et al,2016 (26)	-1	0	0	-1	0	Low
Hoza et al,2015 (27)	-1	0	0	0	0	Moderate
Bustamante et al,2016 (28)	-1	-1	-1	0	0	Very Low
Choi et al,2015 (29)	-1	0	-1	0	0	Low
Chou et al,2017 (30)	-1	0	0	0	0	Moderate
Benzing et al,2019 (31)	-1	-1	0	0	0	Low
Liang et al,2022 (32)	0	0	0	0	0	High
Jensen et al,2004 (33)	-1	0	0	0	0	Moderate
Nejati et al,2021 (34)	-1	-1	0	0	0	Low
Pan et al,2016 (35)	-1	-1	0	0	0	Low
Silva et al,2019 (36)	-1	0	0	0	0	Moderate
Berg et al,2019 (37)	-1	0	0	0	0	Moderate
Hattabi et al,2019 (38)	-1	0	0	0	0	Moderate
Rezaei et al,2018 (39)	0	0	0	0	0	High
Chang et al,2022 (40)	0	-0	0	0	0	High
Li et al,2025 (41)	-1	0	0	0	0	Moderate

## 3 Results

### 3.1 Results of literature search

**Figure 1:**
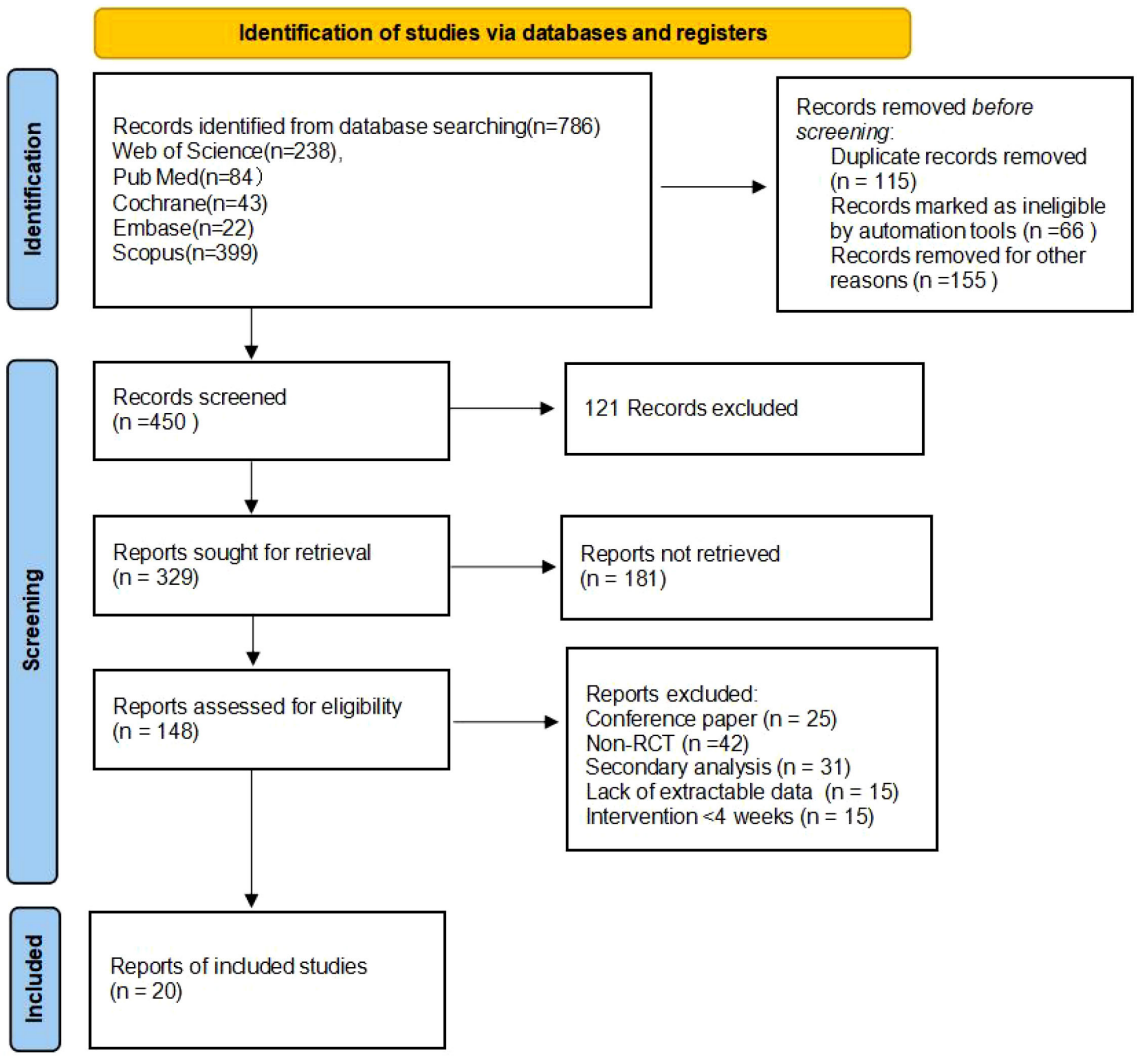
PRISMA flow diagram of the study process.

A total of 789 documents were retrieved from various databases and other sources, 115 documents were excluded after checking, and the remaining 674 were further screened. Eventually, 20 documents were included, covering a total of 1450 subjects, and the screening process is shown in **Figure 1**.

### 3.2 Basic characteristics of the included literature

In the 20 included studies, there were 1450 subjects with an age range of 7–18 years old. The main types of exercise interventions in the experimental group included aerobic exercise, sports games, Exergame, bicycle practice, Taekwondo, yoga, combination sports, table tennis, swimming, and qigong. Intervention cycles covered 4–5 weeks, 6 weeks, 8 weeks, 9–10 weeks, 12 weeks, and lasted at least 20 weeks. The frequency of interventions included 1, 2, 3, and 5 sessions. Intervention duration was 10–31 minutes, 40–50 minutes, 60 minutes, 70 bells, and ≥90 minutes. Intervention intensity was moderate intensity; medium to high intensity. Please see **Table 3** for detailed basic characteristics of the included literature.

**Table 3:** Basic characteristics of included studies.

Author & year	Country	Sample size		Mean age (years)		Instrument	Dose
		E	C	E	C		
Verret et al,2012 (22)	Canada	10	11	9.10 ± 1.1 0		Combination exercise	45min、3times/week、10weeks、moderate intensity
Ziereis et al,2015 (23)	Germany	13/14	16	9.20 ± 1.30	9.5 ± 1.40	Aerobic exercise/Sports games	60min、1times/week、12weeks
Ji et al,2022 (24)	South Korea	16	14	10.50 ± 1.20		Exergame	20min、2times/week、4weeks、 moderate intensity
Kadri et al,2019 (25)	Tunisia	20	20	14.50 ± 3.50	14.20 ± 3.00	Taekwondo	50min、2times/week、5weeks、moderate intensity
Memarmoghaddam et al,2016 (26)	Iran	19	17	8.31 ± 1.29	8.29 ± 1.31	Combination exercise	90min、3times/week、8weeks、moderate intensity
Hoza et al,2015 (27)	United States	94	108	6.83 ± 0.96		Aerobic exercise	31min、5times/week、8weeks、medium to high intensity
Bustamante et al,2016 (28)	United States	19	16	9.40 ± 2.20	8.70 ± 2.00	Sports games	90min、5times/week、10weeks、moderate intensity
Choi et al,2015 (29)	South Korea	13	17	15.80 ± 1.70	16.0 0± 1.20	Aerobic exercise	90min、3times/week、6weeks、moderate intensity
Chou et al,2017 (30)	China	25	25	10.71 ± 1.00	10.30 ± 1.07	Yoga	40min、2times/week、8weeks、moderate intensity
Benzing et al,2019 (31)	Switzerland	28	23	10.46 ± 1.30	10.39 ± 1.44	Exergaming	30min、3times/week、8weeks
Liang et al,2022 (32)	China	40	40	8.37 ± 1.42	8.29 ± 1.27	Combination exercise	60min、3times/week、12weeks、medium to high intensity
Jensen et al,2004 (33)	Australia	11	8	10.63 ± 1.78	9.35 ± 1.70	Yoga	60min、1times/week、20weeks、moderate intensity
Nejati et al,2021 (34)	Iran	15	15	9.43 ± 1.43		Combination exercise	40-50min、3times/week、4-5weeks、moderate intensity
Pan et al,2016 (35)	China	16	16	8.93 ± 1.49	8.87 ± 1.56	Table tennis	70min、2times/week、12weeks、moderate intensity
Silva et al,2019 (36)	Brazil	18	15	12.00 ± 2.00	12.00 ± 1.00	Swimming	45min、2times/week、8weeks、moderate intensity
Berg et al,2019 (37)	Netherlands	263	249	10.50± 1.30		Exergaming	10min、5times/week、9weeks、medium to high intensity
Hattabi et al,2019 (38)	Tunisia	20	20	9.95 ± 1.31	9.75 ± 1.33	Swimming	90min、3times/week、12weeks、moderate intensity
Rezaei et al,2018 (39)	Iran	7	7	9.10 ± 1.30		Yoga	45min、3times/week、8weeks、moderate intensity
Chang et al,2022 (40)	China	16	16	8.31 ± 1.30	8.38 ± 1.20	Table tennis	60min、3times/week、12weeks、moderate intensity
Li et al,2025 (41)	China	60	60	8.40 ± 1.30		Qigong	30min、3times/week、12weeks、medium to high intensity

E, Experimental group; C, Control group.

### 3.3 Evaluation of the quality of the included literature

**Figure 2:**
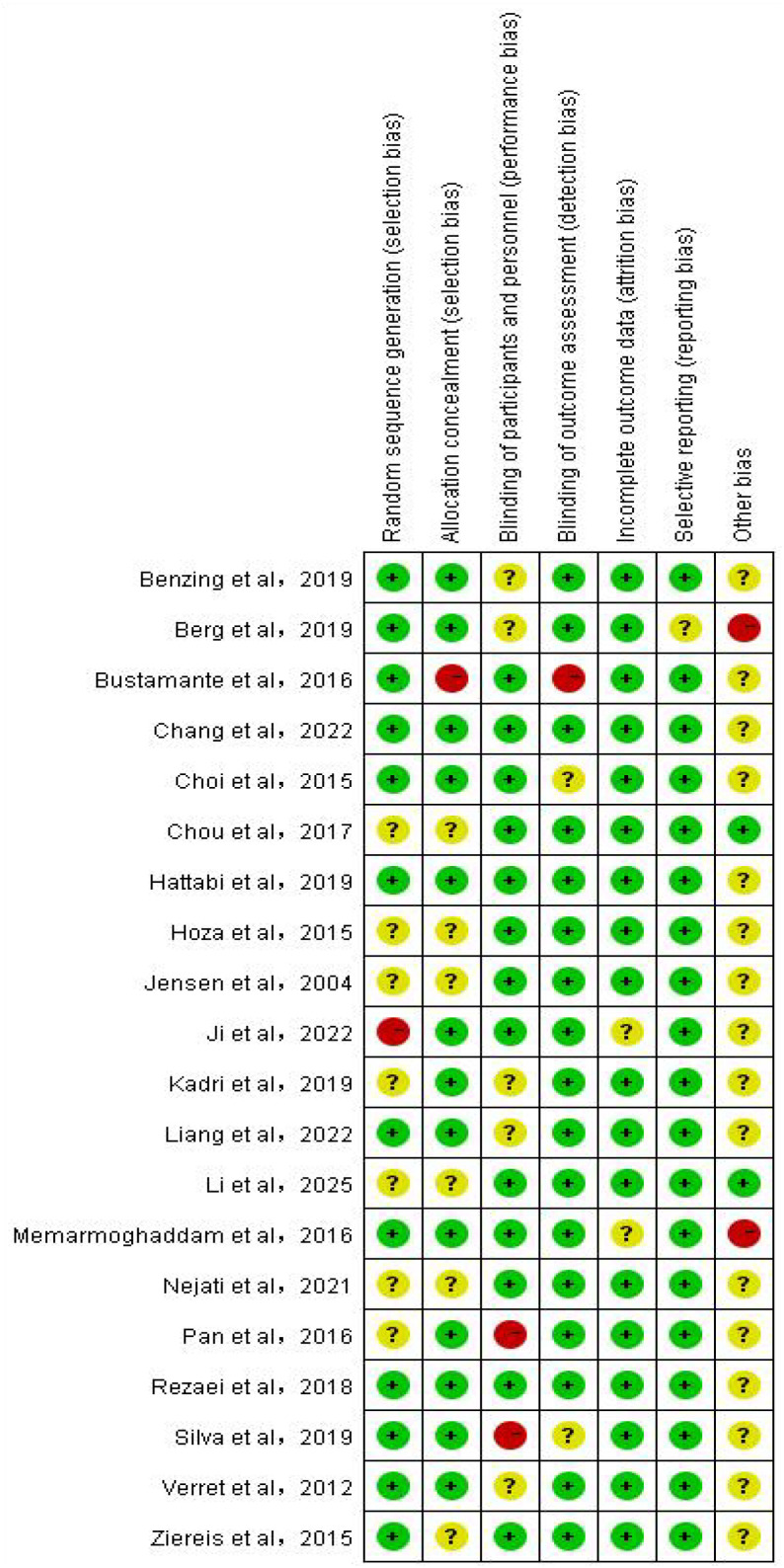
Bias risk diagram for each item.

**Figure 3:**
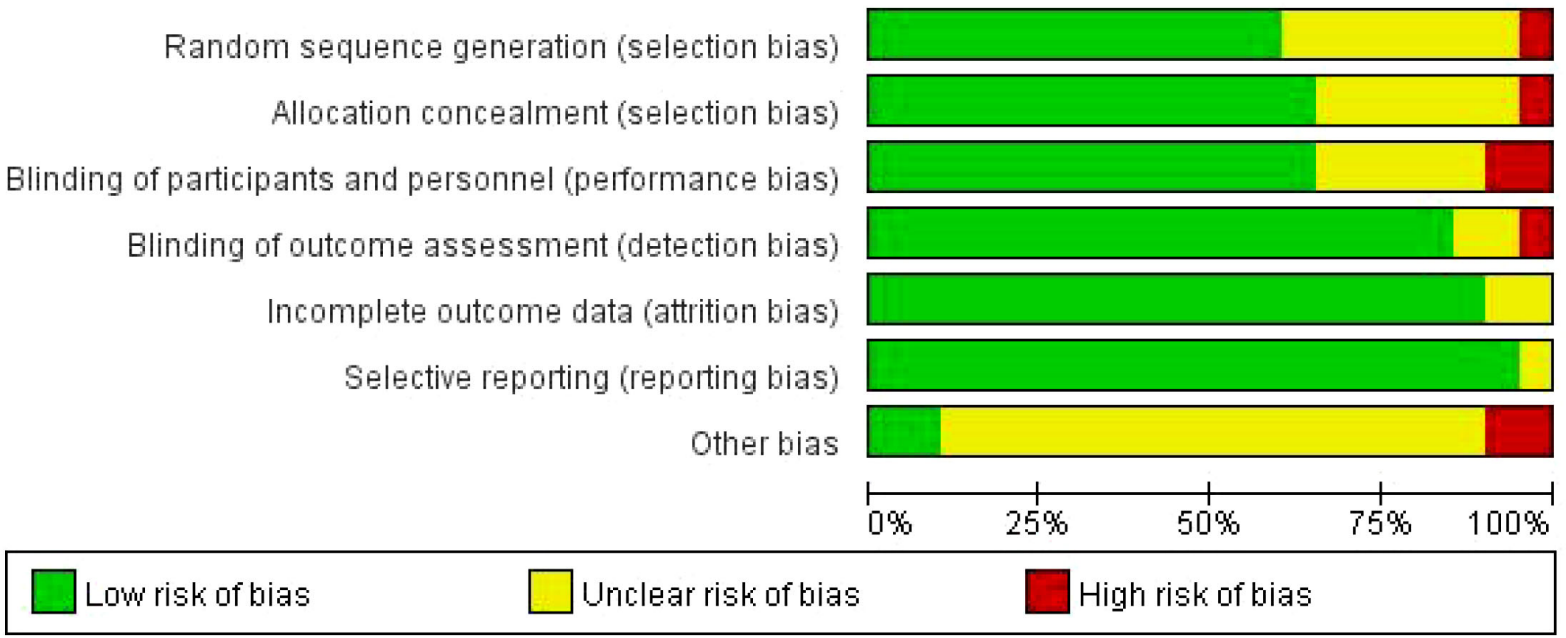
Overall bias risk diagram.

RevMan 5.4 software and the Cochrane Risk of Bias Assessment Tool were used as criteria for a comprehensive and rigorous assessment of the quality of the literature included in the analysis (42). The results of the Cochrane risk of bias assessment for each study are displayed in **Figure 2**, while **Figure 3** visualizes the overall distribution of risk of bias. Studies were classified as low risk in the selection bias assessment if they were assigned using randomization. In contrast, studies that did not use randomization or did not report the randomization process were labeled as high risk of bias. The results of the risk of bias assessment for each study are detailed in **Table 4**.

**Table 4:** Risk of bias assessment (n=20).

Author & year	Random sequence generation (selection bias)	Allocation concealment (selection bias)	Blinding of patients and personnel (performancebias)	Blinding of outcome assessment (detection bias)	Incomplete outcome data (attrition bias)	Selective outcome reporting (reporting bias)	Any other bias
Verret et al,2012 (22)	Low	Low	Unclear	Low	Low	Low	Unclear
Ziereis et al,2015 (23)	Low	Unclear	Low	Low	Low	Low	Unclear
Ji et al,2022 (24)	High	Low	Low	Low	Unclear	Low	Unclear
Kadri et al,2019 (25)	Unclear	Low	Unclear	Low	Low	Low	Unclear
Memarmoghaddam et al,2016 (26)	Low	Low	Low	Low	Unclear	Low	High
Hoza et al,2015 (27)	Unclear	Unclear	Low	Low	Low	Low	Unclear
Bustamante et al,2016 (28)	Low	High	Low	High	Low	Low	Unclear
Choi et al,2015 (29)	Low	Low	Low	Unclear	Low	Low	Unclear
Chou et al,2017 (30)	Unclear	Unclear	Low	Low	Low	Low	Low
Benzing et al,2019 (31)	Low	Low	Unclear	Low	Low	Low	Unclear
Liang et al,2022 (32)	Low	Low	Unclear	Low	Low	Low	Unclear
Jensen et al,2004 (33)	Unclear	Unclear	Low	Low	Low	Low	Unclear
Nejati et al,2021 (34)	Unclear	Unclear	Low	Low	Low	Low	Unclear
Pan et al,2016 (35)	Unclear	Low	High	Low	Low	Low	Unclear
Silva et al,2019 (36)	Low	Low	High	Unclear	Low	Low	Unclear
Berg et al,2019 (37)	Low	Low	Unclear	Low	Low	Unclear	High
Hattabi et al,2019 (38)	Low	Low	Low	Low	Low	Low	Unclear
Rezaei et al,2018 (39)	Low	Low	Low	Low	Low	Low	Unclear
Chang et al,2022 (40)	Low	Low	Low	Low	Low	Low	Unclear
Li et al,2025 (41)	Unclear	Unclear	Low	Low	Low	Low	Low

### 3.4 Results of reticulation meta-analysis

#### 3.4.1 Reticulation Meta-analysis

**Figure 4:**
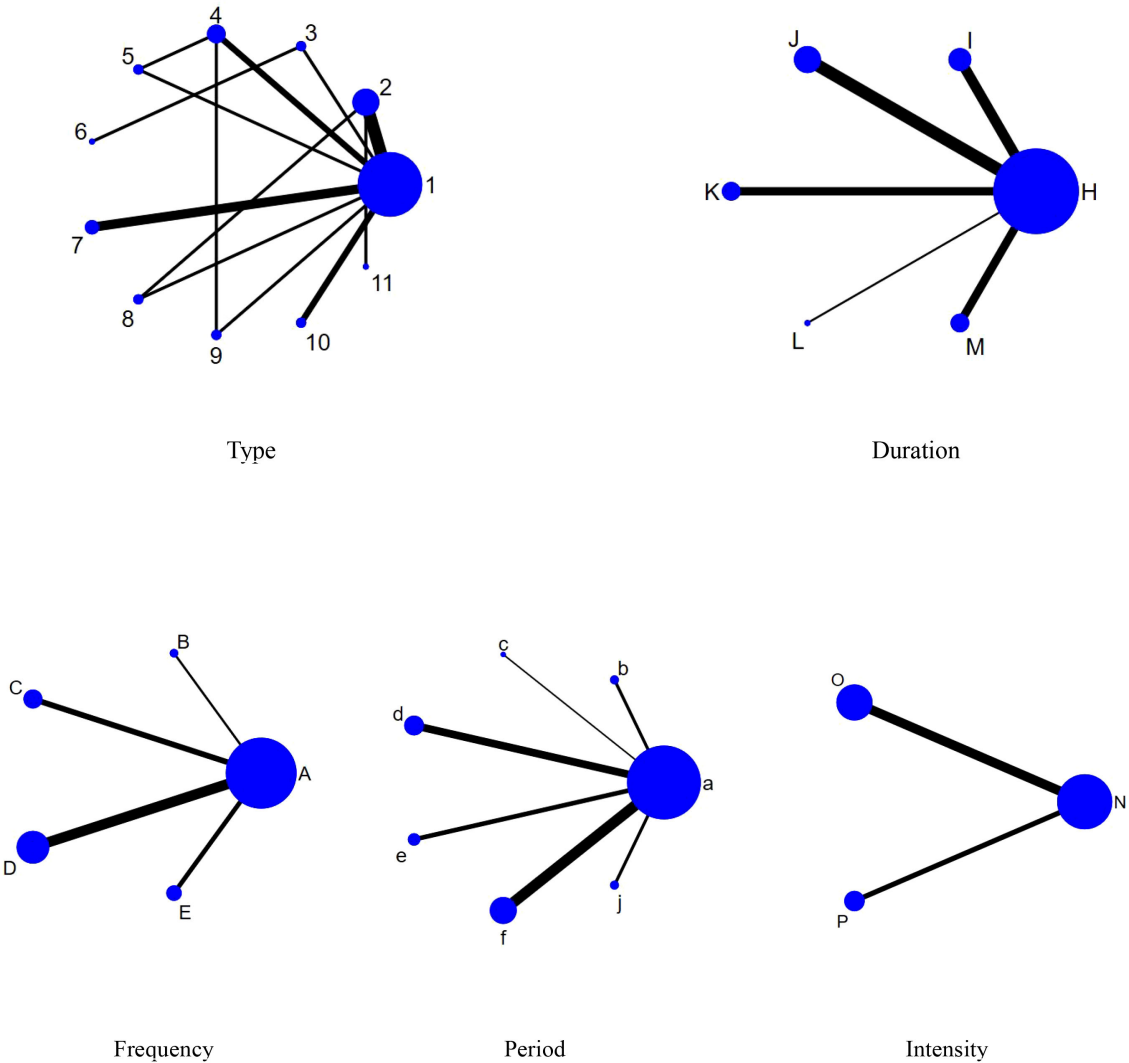
Network evidence diagram. 1,con;2,Aerobic exercise;3,Sports games;4,Exergame;5,Cycling exercise;6,Taekwondo;7,Yoga;8,Combination exercise;9,Table tennis;10,Swimming;11,Qigong; A,con;B,1times;C,2times;D,3times;E,5times;H,con;I,10-31min;J,40-50min;K,60min;L,70min;M,≥90min;a,con;b,4-5weeks;c,6weeks;d,8weeks;e,9-10weeks;f,12weeks;j,≥20weeks;N,con;O,Moderate intensity;P,Medium to high intensity.

Reticulated Meta-analysis is a method of synthesizing direct and indirect evidence that allows the effects of more than two interventions to be compared simultaneously. In a reticulation graph, each node represents an intervention, the size of the area of the node reflects the sample size, and the thickness of the lines connecting the nodes indicates the number of included studies. See **Figure 4** for a specific reticulation evidence map.

#### 3.4.2 Results of two-by-two comparisons between elements of exercise prescription

**Figure 5:**
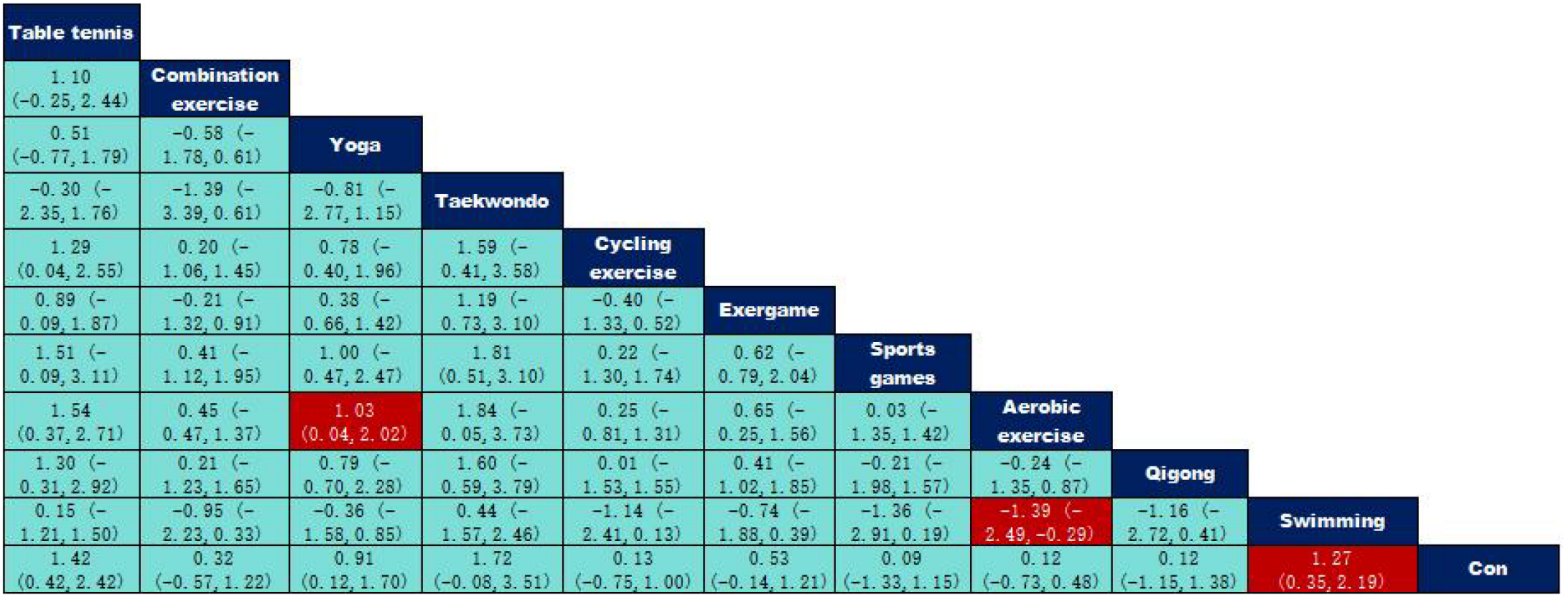
League table of pairwise comparisons of intervention effects among exercise type elements.

**Figure 6:**
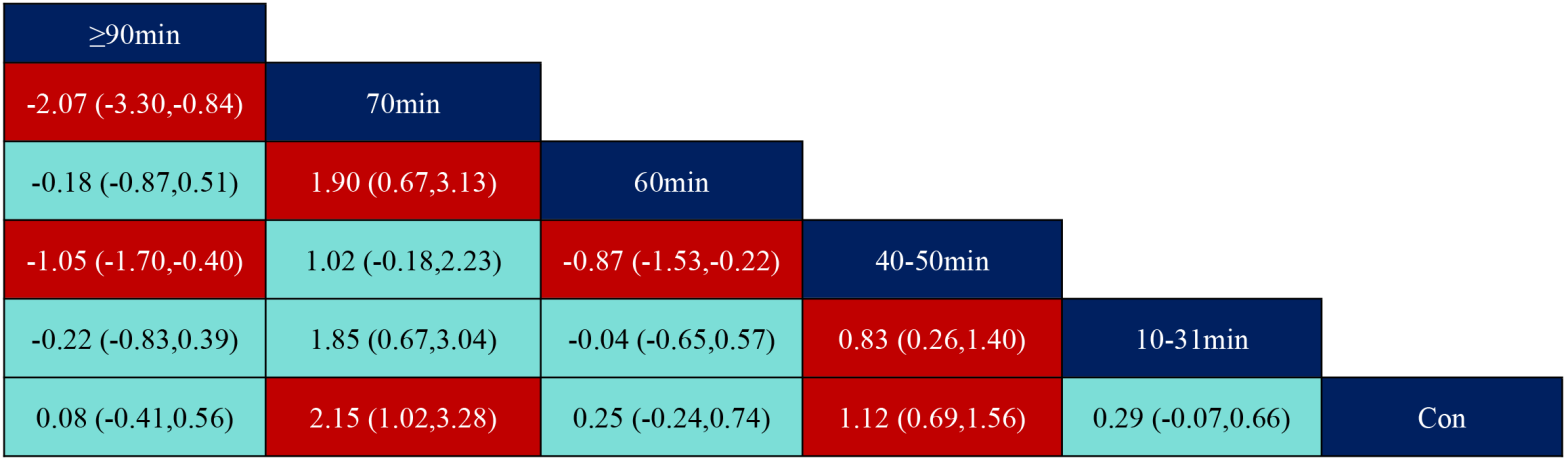
League table of pairwise comparisons of intervention effects among exercise duration elements.

**Figure 7:**
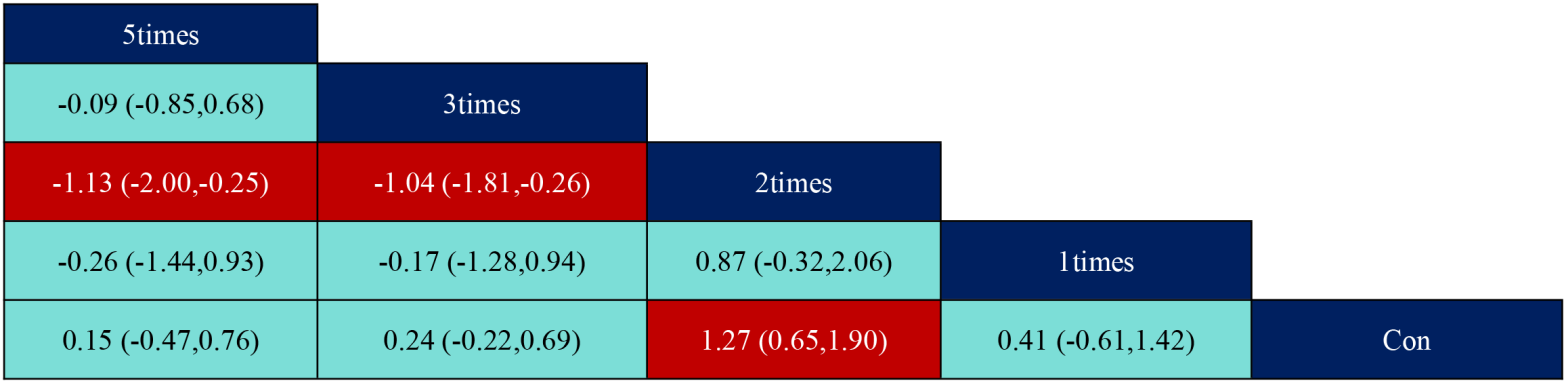
League table of pairwise comparisons of intervention effects among exercise frequency elements.

**Figure 8:**
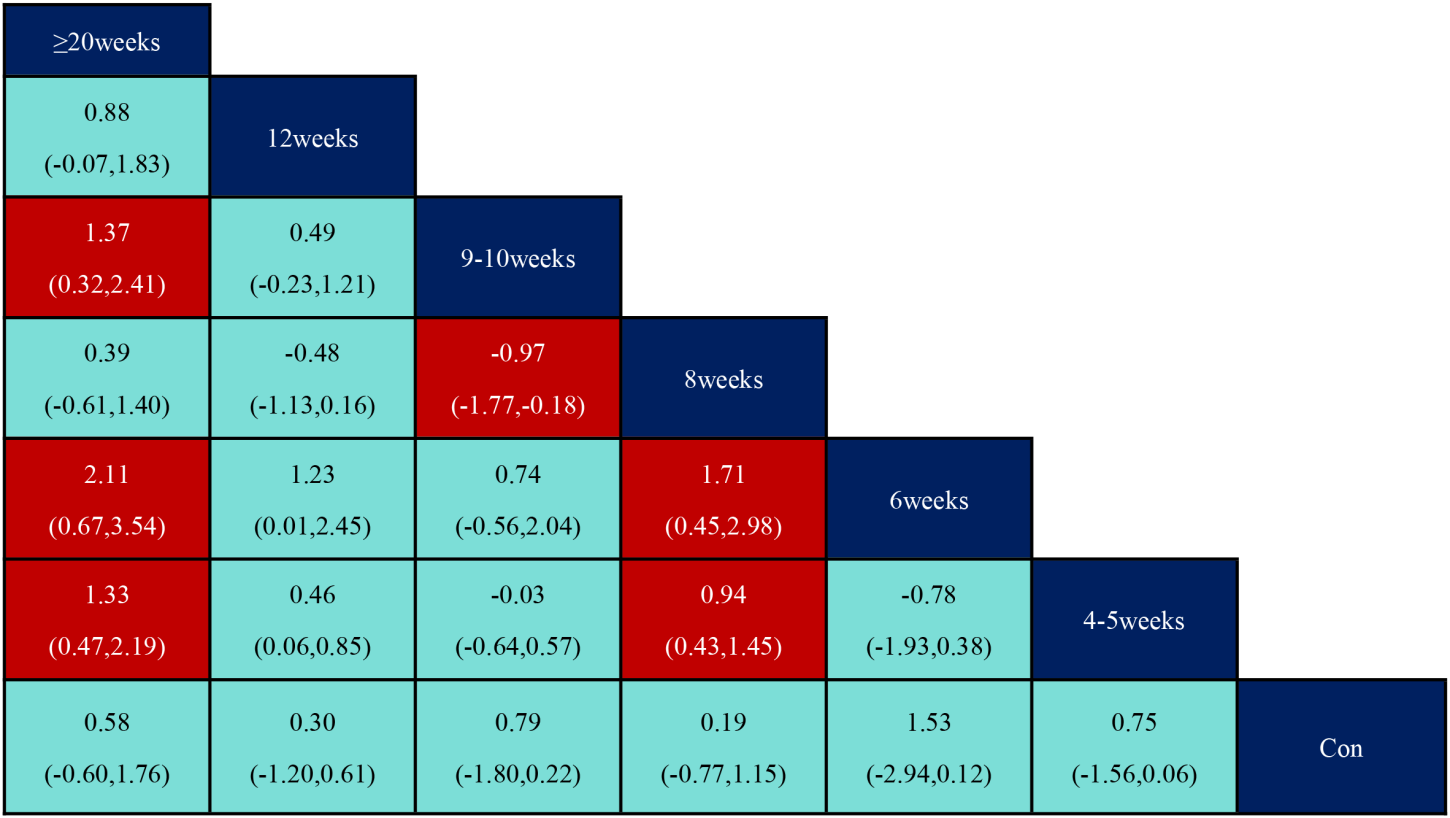
League table of pairwise comparisons of intervention effects among exercise period elements.

**Figure 9:**
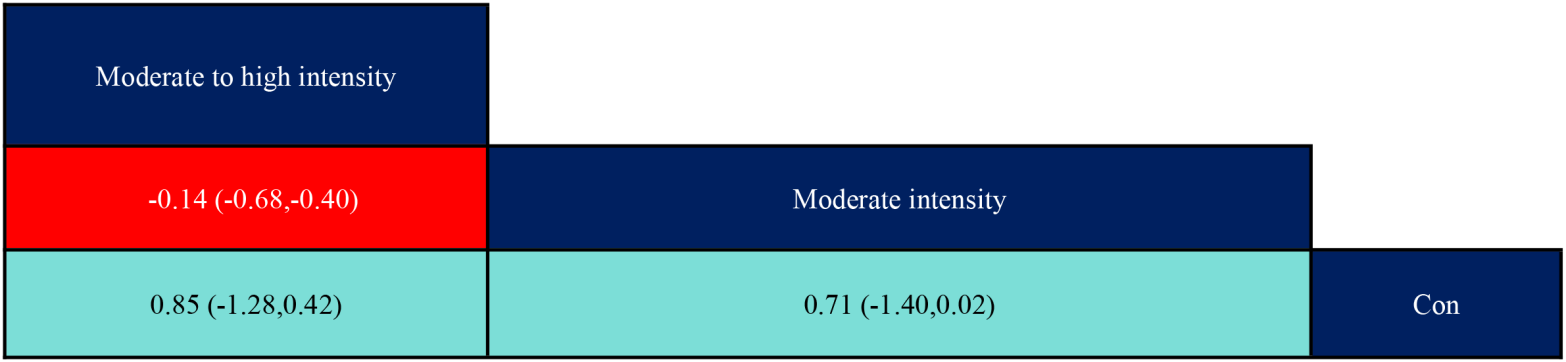
League table of pairwise comparisons of intervention effects among exercise intensity elements. The red numbers are statistically significant.

According to the data in **Figure 5**, swimming (SMD=1.27, 95% CI: (0.35,2.19)), p<0.05 was significantly better than the control group, while aerobic exercise (SMD=-1.39, 95% CI: (-2.49,-0.29)) was significantly less effective than swimming exercise intervention, and yoga (SMD=1.03, 95% CI: (0.04,2.02)) was significantly better than aerobic exercise intervention. According to the data in **Figure 6** the intervention duration of 40–50 minutes (SMD=1.12, 95% CI: (0.69,1.56)) and 70 minutes (SMD=2.15, 95% CI (1.02,3.28)) was significantly better than the control group. Whereas the intervention effect minutes of 40–50 minutes (SMD=0.83, 95% CI: (0.26,1.40)) was significantly better than the intervention effect minutes of 10–31 minutes, the intervention effect minutes of 60 minutes (SMD=-0.87, 95% CI: (-1.53,-0.22)) was significantly lower than the intervention effect minutes of 40–50 minutes, and the intervention effect minutes of 70 minutes (SMD=1.90,95%CI: (0.67,3.13)) intervention effect was significantly better than the 60-minute intervention effect, and the ≥90-minute (SMD=-2.07,95%CI (-3.30,-0.84)) intervention effect was significantly lower than the 70-minute intervention effect and the 60-minute (SMD=-1.05,95%CI (-1.70,-0.40)) intervention Effect. According to the data in **Figure 7** the intervention effect of 2 times per week (SMD=1.27, 95% CI: (0.65,1.90)) was significantly better than that of the control group, whereas the intervention effect of 3 times per week (SMD=-1.04, 95% CI (-1.81,-0.26)) was significantly lower than that of 2 times per week, and that of 5 times per week (SMD=-1.13, 95% CI (-2.00, -0.25)) was significantly lower than that of 2 times per week. 2.00, -0.25)) had a significantly lower intervention effect than 3 times. According to the data in **Figure 8**, the intervention effect of an intervention period of 8 weeks was significantly better than the intervention effect of 6 weeks (SMD=1.71, 95% CI: (0.45,2.98)) and 4–5 weeks (SMD=0.94, 95% CI: (0.43,1.45)), and the intervention effect of 9–10 weeks (SMD=-0.97, 95% CI (-1.77,- 0.18)) was significantly lower than the intervention effect of 8 weeks, and the intervention effect of lasting at least 20 weeks (SMD=1.37, 95% CI(0.32,2.41)) was significantly better than the intervention effect of 12 weeks, as well as the intervention effect of 6 weeks (SMD=2.11, 95% CI(0.67,3.54)) and the intervention effect of 4–5 weeks (SMD=1.33, 95% CI (0.47,2.19)) intervention effects. According to the data in **Figure 9**, the intervention effect of medium to high intensity was significantly lower than that of medium intensity (SMD=-0.14, 95% CI (-0.68, -0.40)), and there was no significant difference in the other two-by-two comparisons.

#### 3.4.3 Probabilistic ranking of optimal interventions for each element of exercise prescription

**Figure 10:**
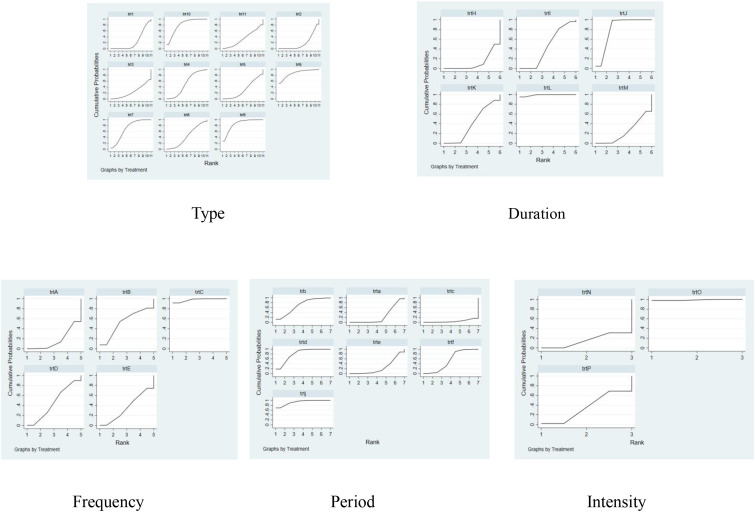
Probability ranking chart of dose-response effects of exercise prescription elements. 1,con;2,Aerobic exercise;3,Sports games;4,Exergame;5,Cycling exercise;6,Taekwondo;7,Yoga;8,Combination exercise;9,Table tennis;10,Swimming;11,Qigong; A,con;B,1times;C,2times;D,3times;E,5times;H,con;I,10-31min;J,40-50min;K,60min;L,70min;M,≥90min;a,con;b,4-5weeks;c,6weeks;d,8weeks;e,9-10weeks;f,12weeks;j,≥20weeks;N,con;O,Moderate intensity;P,Medium to high intensity.

According to SUCRA from **Table 5** and **Figure 10**, the order of the effects of different types of exercise interventions on the participants with ADHD was: Taekwondo (SUCRA=87.1) > Table tennis (SUCRA=85.2) > Swimming (SUCRA=80.5) > Yoga (SUCRA=69.8) > Exergame (SUCRA=52.1) > Combination exercise (SUCRA=43.6) > Qigong (SUCRA=34.1) > Qigong (SUCRA=34.1) > Qigong (SUCRA=34.1) Swimming (SUCRA=80.5)>Yoga (SUCRA=69.8)>Exergame (SUCRA=52.1)>Combination exercise (SUCRA=43.6)>Qigong (SUCRA=34.1)>Cycling exercise (SUCRA=31.5)>Sports games (SUCRA=23.9). Aerobic exercise (SUCRA=23.8)>Con (SUCRA=18.4).

**Table 5:** SUCRA values for the efficacy of interventions of each element of exercise prescription.

Rank	Type	SUCRA	Duration	SUCRA	Frequency	SUCRA	Period	SUCRA	Intesity	SUCRA
1	Taekwondo	87.1	70min	99	2times	97.8	≥20weeks	92.5	Moderate intensity	98.9
2	Table tennis	85.2	40-50min	80.8	1times	53.6	8weeks	80.2	Medium to high intensity	35.6
3	Swimming	80.5	10-31min	45.1	3times	45.5	4-5weeks	68.9	Con	15.6
4	Yoga	69.8	60min	39.8	5times	35.8	12weeks	54		
5	Exergame	52.1	≥90min	23.6	Con	17.8	9-10weeks	25.3		
6	Combination exercisen	43.6	Con	11.7			6weeks	24.4		
7	Qigong	34.1					Con	4.6		
8	Cycling exercise	31.5								
9	Sports games	23.9								
10	Aerobic exercise	23.8								
11	Con	18.4								

The ranking of the effects of different exercise intervention durations on the intervention effects on participants with ADHD was: 70min (SUCRA=99.0) > 40- 50min (SUCRA=80.8) > 10- 31min (SUCRA=45.1) > 60min (SUCRA=39.8) > ≥90min (SUCRA=23.6) > Con (SUCRA=11.7).

The effects of different exercise intervention frequencies on participants with ADHD were ranked as follows: 2 times (SUCRA=97.8) > 1 time (SUCRA=53.6) > 3 times (SUCRA=45.5) > 5 times (SUCRA=35.8) > Con (SUCRA=17.8).

The rank order of the effect of different exercise intervention cycles on the effect of intervention on participants with ADHD was: ≥ 20 weeks (SUCRA=92.5) > 8 weeks (SUCRA=80.2) > 4–5 weeks (SUCRA=68.9) > 12 weeks (SUCRA=54) > 9–10 weeks (SUCRA=25.3) > 6 weeks (SUCRA=24.4) > Con (SUCRA=4.6).

The ranking of the effects of different exercise intervention intensities on the effects of the intervention on participants with ADHD was Moderate intensity (SUCRA=98.9) > Medium to high intensity (SUCRA=35.6) > Con (SUCRA=15.6).

### 3.5 Sensitivity analysis

In this study, a sensitivity analysis was conducted to investigate the sources of heterogeneity using the leave-one-out method (43). The results of the analysis showed that the fluctuation range of the combined effect value remained between 0.05 and 0.95 after the removal of any single literature. This indicates that the deletion of each piece of literature has a more limited impact on the overall combined effect value, suggesting that the analytical results of this study have a high degree of stability and reliability. Therefore, it can be confirmed that the conclusions obtained are highly robust.

### 3.6 Publication bias test

**Figure 11:**
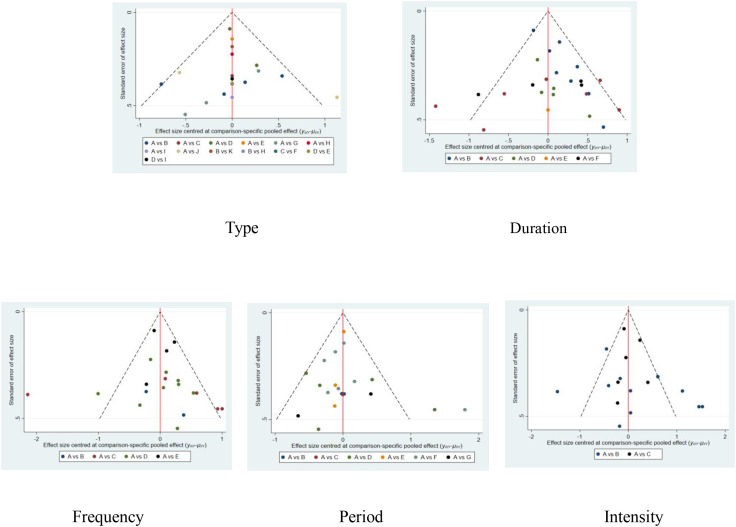
Funnel plot of dose-response effects of exercise prescription elements. 1,con;2,Aerobic exercise;3,Sports games;4,Exergame;5,Cycling exercise;6,Taekwondo;7,Yoga;8,Combination exercise;9,Table tennis;10,Swimming;11,Qigong; A,con;B,1times;C,2times;D,3times;E,5times;H,con;I,10-31min;J,40-50min;K,60min;L,70min;M,≥90min;a,con;b,4-5weeks;c,6weeks;d,8weeks;e,9-10weeks;f,12weeks;j,≥20weeks;N,con;O,Moderate intensity;P,Medium to high intensity.

As can be observed from **Figure 11**, the funnel plot of each indicator is roughly symmetrically distributed, and most of the data points are located in the inner part of the funnel plot, while only a few data points are located in the outer part of the funnel. Overall, the distribution pattern of the funnel plot suggests that there may be a small publication bias in this study. To further verify this, this study assessed publication bias using the Egger test, which showed a p-value of 0.119. This result suggests that no significant publication bias was found (44). However, even though the statistical analysis failed to reveal significant bias, the results of the study need to be interpreted with caution because publication bias may not have been completely ruled out, and other potential factors may still affect the reliability of the results.

## 4 Discussion

Although several systematic reviews and meta-analyses have recently discussed the effects of exercise on interventions for individuals with ADHD (45, 46), our reticulated meta-analysis is the first to discuss the effects of exercise on inhibitory control in individuals with ADHD concerning the components of exercise prescription. In addition, we ranked the magnitude of the effect of different exercise types, exercise frequency, exercise duration, exercise period, and exercise intensity on inhibitory control in children and adolescents with ADHD, which will help to further identify the most effective exercise intervention prescriptions in the population of children and adolescents with ADHD.

The present study found through a net meta-analysis that the best type of exercise to improve inhibitory control in children and adolescents with ADHD may be taekwondo; the best duration of intervention may be 70 min; the best frequency of intervention may be 2 times/week; the best cycle of intervention may be one that lasts for at least 20 weeks; and the best intensity of intervention may be of moderate intensity. The possible reasons for this lie in the theory of brain plasticity, which has been explored by academics in terms of neurophysiological mechanisms (47). This theory suggests that prolonged exercise triggers structural and functional changes in the brain, increasing the capacity of the prefrontal cortex, which in turn affects cognitively active areas of the brain. Notably, inhibitory neurons are heavily distributed in these regions (48). In addition, related studies have also shown that long-term participation in sports has a more significant intervention effect on children and adolescents with ADHD (49).

Taekwondo may be the best type of exercise to improve inhibitory control in youth with ADHD, but given the limited sample size and literature, taekwondo needs to be promoted with caution despite its potential to be the best exercise. This result is somewhat different from the existing Meta-analysis. The results of the established Meta-analysis suggest that ball games may be the type of intervention that has the best impact on youth with ADHD (11). From a neurophysiological perspective, physical activity can directly affect the release of neurotransmitters in the brain, which in turn alters neural circuits (50), especially in populations with ADHD, where brain regions such as the prefrontal cortex and the basal ganglia are often underfunctioning and accompanied by deficiencies of neurotransmitters, such as dopamine and norepinephrine, which are essential for inhibitory control (51), and the effect of different types of exercise on the neurotransmission systems vary (52, 53). Taekwondo, on the other hand, as an offensive and defensive sport, involves body coordination, concentration, and self-control, which not only provides a physical exercise effect, but also an exercise for the brain (54). Taekwondo training requires children and adolescents to be highly concentrated in executing movements, especially when performing precise kicking, fighting, and defending, which requires the participants’ brains to make quick judgments and decisions in a short period of time (55). At this time, increased activity in the prefrontal cortex promotes the release of dopamine and norepinephrine, which in turn improves the brain’s ability to control attention and inhibit impulses (56).

In addition, the present study also explored the effect of intervention duration on inhibitory control in youth with ADHD, and the duration of 70 minutes per intervention may be optimal, a finding that is consistent with the results of an existing meta-analysis, which also pointed out that a prolonged exercise intervention each time had a positive effect on improving inhibitory control in youth with ADHD (57). According to research, interventions of moderate exercise duration can help children and adolescents maintain high levels of attention and help to improve self-control after exercise (58). 70-minute exercise interventions may have the best intervention effect on inhibitory control in youth with ADHD, which may be closely related to the temporal characteristics of neurotransmitter release in the brain (59). Studies have shown that exercise lasts 45 to 60 minutes to significantly increase dopamine and norepinephrine neurotransmitter release (60). And concentrations of these neurotransmitters peak between 15 and 30 minutes after exercise (61). Thus, the 70-minute exercise duration coincides with two critical periods of neurotransmitter release: the first 60 minutes of exercise activate transmitter release, and the next 10 minutes is the period of peak transmitter concentration when training maximizes the effects of inhibitory control (62, 63). In contrast, short durations of 40 to 50 min of exercise, while capable of triggering neurotransmitter release, release relatively small amounts that are insufficient to fully activate relevant receptor pathways in the prefrontal cortex of the brain (64). Although a 60-minute exercise duration can satisfy the demand for neurotransmitter release, the lack of a maintenance phase of peak transmitter concentration can lead to a reduced neuroplasticity effect, while on the other hand, it has been shown that a prolonged 90-minute exercise period may have led to a significant increase in cortisol concentration, a change that inhibits the efficiency of dopamine synthesis through a negative feedback mechanism (65).

Regarding intervention frequency, the present study found that an intervention frequency of 2 times per week was the most effective, which is consistent with the results of existing studies (57, 66, 67). Multiple studies have shown that appropriate intervention frequency is critical for inhibitory control in children with ADHD; in other words, regular exercise twice a week is most effective in improving executive functioning (e.g., intervening to inhibit impulses) in children and adolescents with ADHD (28, 57). Too high a frequency of intervention may lead to overexertion and low energy, whereas too low a frequency may lead to a lag in the effects of the intervention and affect the adaptive response of the nervous system. Another study showed that an intervention frequency of 2 times per week was effective in balancing neurostimulation and the physical load of children and adolescents, ensuring that they did not react negatively to overtraining, and avoiding physical fatigue and psychological overstress (68). At the same time, the frequency of 2 times per week provides sufficient time for children and adolescents with ADHD to recover and consolidate the last training session (69).

The present study found that intervention cycles lasting at least 20 weeks may be the best option for the treatment of attention deficit hyperactivity disorder (ADHD), which is consistent with existing meta-analyses that have found that long-term interventions can help to consolidate the treatment effects and increase the degree of neuroadaptation and behavioral improvement in children (70). Longer interventions provide more opportunities for neuroadaptation, allowing for gradual neurological improvements over a longer period, and the positive effects that long-term exercise interventions can have on children and adolescents with ADHD (71). It has also been shown that exercise interventions lasting more than 6 months can significantly improve attention, impulse control, and task performance in children and adolescents with ADHD (40). Long-term exercise intervention improves the efficiency of signaling in neural circuits, particularly in the prefrontal cortex, a key region of the brain responsible for higher cognitive functions, and its improved functioning significantly enhances self-control and concentration in children and adolescents (7).

The present study found that a moderate-intensity intervention may be most beneficial for improving inhibitory control in children and adolescents with attention deficit hyperactivity disorder (ADHD). This finding differs from a previous meta-analysis, which concluded that high-intensity sports training is more appropriate for improving inhibitory control in children and adolescents with ADHD (72). In contrast, studies have shown that moderate-intensity sport significantly improves core symptoms, including inhibitory control, attentional focus, and executive functioning, in children and adolescents with ADHD (73). Moderate-intensity exercise is typically defined as an activity between light and high intensity, characterized by a significant increase in heart rate and respiratory rate, but not yet at a vigorous level. As defined by the American College of Sports Medicine (ACSM), moderate-intensity exercise typically corresponds to exercise intensities in the range of 40% to 59% of maximal oxygen uptake reserve (VO_2_R) (74). Although exercise interventions are overall beneficial for children and adolescents with ADHD, too much high-intensity exercise may be counterproductive. Studies have found that improvements in cognitive performance are reduced after vigorous exercise compared to moderate-intensity exercise in children and adolescents with ADHD (73). Another experiment on the effects of acute aerobic exercise at different intensities showed that only after low- and moderate-intensity exercise, the response rate of executive functions (e.g., Flanker inhibition task) was significantly faster in children and adolescents with ADHD than after vigorous exercise (73). In addition, the psychological dimension cannot be ignored. High-intensity training is often taxing and even frustrating for children and adolescents and may lead to decreased self-efficacy, meaning that children feel less confident in their ability to complete the training and control their behavior. Children and adolescents with ADHD are inherently characterized by inattention and poor control, and if the intensity and complexity of exercise exceeds the ability of the child or adolescent, the child is likely to be resistant, which is not conducive to long-term adherence (6). Therefore, moderate-intensity exercise interventions are gaining attention and can be used to help children and adolescents with ADHD improve their symptoms.

## 5 Limitations

Firstly, the included literature mainly focused on exercise intervention without strict control of daily diet and other activities, and the influence of potential moderating factors (e.g., age, ADHD subtype, medication use, etc.) on the relationship between exercise and inhibitory control was not sufficiently considered; secondly, no distinction was made between genders, and children and adolescents with ADHD of different genders may respond differently to intervention due to the developmental characteristics of puberty; the issue of potential publication bias that would The issue of potential publication bias affecting the credibility of the results was also not explored in depth; finally, the literature search was limited to English, and other languages were not searched, and the inclusion of less literature on qigong, taekwondo, and sports games affected the comprehensiveness of the included literature to a certain extent.

## 6 Implications for research

This study provides important theoretical support for exercise interventions for children and adolescents with ADHD, revealing the effectiveness of exercise interventions in improving behavioral regulation. The findings suggest that exercise type, duration, frequency, periodization, and intensity have a significant effect on inhibitory control in children and adolescents with ADHD, particularly in terms of the neurophysiological mechanisms at play. This provides an intervention framework for future research.

## 7 Implications for clinical practice

This study demonstrates the importance of exercise interventions in the treatment of children and adolescents with ADHD, particularly the need to tailor the selection of exercise type and intervention duration to individual patient differences. Based on the results of this study, clinical staff can develop a personalized exercise treatment plan that takes into account the individual characteristics of children and adolescents with ADHD (e.g., age, gender, and interest in exercise). At the same time, studies have shown that exercise is not only physical exercise, but its effect on the neural function of the brain cannot be ignored, which provides a new perspective for the integrated treatment of ADHD in clinical practice. In addition, future clinical studies could combine exercise intervention with medication to improve treatment effects and reduce the risk of drug dependence.

## 8 Conclusion

This study assessed the impact of different exercise prescriptions on the effectiveness of ADHD interventions by including 20 studies and using network Meta-analysis. The available evidence suggests that specific exercise prescriptions, such as taekwondo intervention, 70 minutes per intervention duration, 2 times per week intervention frequency, intervention cycles longer than 20 weeks, and moderate-intensity exercise, may have the best effect on improving inhibitory control in children and adolescents with ADHD. However, we should also be concerned about individual differences in children’s expression of ADHD symptoms, and each child’s specific symptoms need to be accurately assessed before the intervention is implemented. Meanwhile, due to the relatively limited number of included studies, the generalizability and reliability of these findings still need to be further verified. Nevertheless, this study provides a valuable reference for the clinical development of individualized exercise intervention programs. Future studies should expand the sample size, explore the effects of different exercise prescriptions on different ADHD subtypes, and enhance the evaluation of long-term intervention effects. At the same time, when implementing exercise interventions in the clinic or in the school, it is necessary to pay attention to potential barriers such as resource constraints, adherence challenges, and the need for training in specific sports (e.g., taekwondo), and to address them appropriately to ensure that the interventions are carried out smoothly and achieve good results.

## Data Availability

The original contributions presented in the study are included in the article/supplementary material. Further inquiries can be directed to the corresponding author.

## References

[1] Raman SR Man KK Bahmanyar S Berard A Bilder S Boukhris T . Trends in attention-deficit hyperactivity disorder medication use: A retrospective observational study using population-based databases. Lancet Psychiatry. (2018) 5:824–35. doi: 10.1016/S2215-0366(18)30293-1 30220514

[2] Wilens TE Biederman J Faraone SV Martelon M Westerberg D Spencer TJ . Presenting adhd symptoms, subtypes, and comorbid disorders in clinically referred adults with adhd. J Clin Psychiatry. (2009) 70:15333. doi: 10.4088/JCP.08m04785pur PMC2948439 20031097

[3] Schachar RJ Tannock R Logan G . Inhibitory control, impulsiveness, and attention deficit hyperactivity disorder. Clin Psychol Rev. (1993) 13:721–39. doi: 10.1016/S0272-7358(05)80003-0

[4] Roselló B Berenguer C Baixauli I Mira Á Martinez-Raga J Miranda A . Empirical examination of executive functioning, adhd associated behaviors, and functional impairments in adults with persistent adhd, remittent adhd, and without adhd. BMC Psychiatry. (2020) 20:1–12. doi: 10.1186/s12888-020-02542-y 32204708 PMC7092442

[5] Graham J Banaschewski T Buitelaar J Coghill D Danckaerts M Dittmann RW . European guidelines on managing adverse effects of medication for adhd. Eur Child Adolesc Psychiatry. (2011) 20:17–37. doi: 10.1007/s00787-010-0140-6 21042924 PMC3012210

[6] Chan Y-S Jang J-T Ho C-S . Effects of physical exercise on children with attention deficit hyperactivity disorder. Biomed J. (2022) 45:265–70. doi: 10.1016/j.bj.2021.11.011 PMC9250090 34856393

[7] Christiansen L Beck MM Bilenberg N Wienecke J Astrup A Lundbye-Jensen J . Effects of exercise on cognitive performance in children and adolescents with adhd: potential mechanisms and evidence-based recommendations. J Clin Med. (2019) 8:841. doi: 10.3390/jcm8060841 31212854 PMC6617109

[8] Radel R Brisswalter J Perrey S . Saving mental effort to maintain physical effort: A shift of activity within the prefrontal cortex in anticipation of prolonged exercise. Cognitive Affective Behav Neurosci. (2017) 17:305–14. doi: 10.3758/s13415-016-0480-x 27858329

[9] Subcommittee on Attention-Deficit/Hyperactivity Disorder SCoQI, Management . Adhd: Clinical Practice Guideline for the Diagnosis, Evaluation, and Treatment of Attention-Deficit/Hyperactivity Disorder in Children and Adolescents. IL, USA: American Academy of Pediatrics Elk Grove Village (2011) p. 1007–22. 10.1542/peds.2011-2654 PMC4500647 22003063

[10] Tremblay MS Chaput J-P Adamo KB Aubert S Barnes JD Choquette L . Canadian 24-hour movement guidelines for the early years (0–4 years): an integration of physical activity, sedentary behaviour, and sleep. BMC Public Health. (2017) 17:1–32. doi: 10.1186/s12889-017-4859-6 29219102 PMC5773896

[11] Li H Zhang P Yan B . Does type of exercise matter? Network meta-analysis of the effects of different exercise modalities on inhibitory control in children with attention deficit hyperactivity disorder. Curr Psychol. (2024) 17:1–10. doi: 10.1007/s12144-023-04703-0

[12] Billinger SA Boyne P Coughenour E Dunning K Mattlage A . Does aerobic exercise and the fitt principle fit into stroke recovery? Curr Neurol Neurosci Rep. (2015) 15:1–8. doi: 10.1007/s11910-014-0519-8 PMC4560458 25475494

[13] Yang Y Wu C-H Sun L Zhang T-R Luo J . The impact of physical activity on inhibitory control of adult adhd: A systematic review and meta-analysis. J Global Health. (2025) 15:04025. doi: 10.7189/jogh.15.04025 PMC11907377 40084538

[14] Amatriain-Fernández S Ezquerro García-Noblejas M Budde H . Effects of chronic exercise on the inhibitory control of children and adolescents: A systematic review and meta-analysis. Scandinavian J Med Sci Sports. (2021) 31:1196–208. doi: 10.1111/sms.13934 33559271

[15] Zhu F Zhu X Bi X Kuang D Liu B Zhou J . Comparative effectiveness of various physical exercise interventions on executive functions and related symptoms in children and adolescents with attention deficit hyperactivity disorder: A systematic review and network meta-analysis. Front Public Health. (2023) 11:1133727. doi: 10.3389/fpubh.2023.1133727 37033046 PMC10080114

[16] Gianola S Castellini G Andreano A Corbetta D Frigerio P Pecoraro V . Effectiveness of treatments for acute and sub-acute mechanical non-specific low back pain: protocol for a systematic review and network meta-analysis. Systematic Rev. (2019) 8:1–8. doi: 10.1186/s13643-019-1116-3 PMC6688358 31395091

[17] Hutton B Salanti G Caldwell DM Chaimani A Schmid CH Cameron C . The prisma extension statement for reporting of systematic reviews incorporating network meta-analyses of health care interventions: checklist and explanations. Ann Internal Med. (2015) 162:777–84. doi: 10.7326/M14-2385 26030634

[18] Munn Z Stern C Aromataris E Lockwood C Jordan Z . What kind of systematic review should I conduct? A proposed typology and guidance for systematic reviewers in the medical and health sciences. BMC Med Res Method. (2018) 18:1–9. doi: 10.1186/s12874-017-0468-4 PMC5761190 29316881

[19] Idris SA Qureshi AG Elkhair IS Idris TA Adam AM Mohammed NK . Usefulness of endnote software for writing scientific manuscripts: A comparative study. Health Res. (2019) 7:6–10. doi: 10.4314/sjms.v4i1.44876

[20] Sterne JA Savović J Page MJ Elbers RG Blencowe NS Boutron I . Rob 2: A revised tool for assessing risk of bias in randomised trials. bmj. (2019) 28:366. doi: 10.1136/bmj.l4898 31462531

[21] Shim S Yoon B-H Shin I-S Bae J-M . Network meta-analysis: application and practice using stata. Epidemiol Health. (2017) 39:e2017047. doi: 10.4178/epih.e2017047 29092392 PMC5733388

[22] Verret C Guay M-C Berthiaume C Gardiner P Béliveau L . A physical activity program improves behavior and cognitive functions in children with adhd: an exploratory study. J attention Disord. (2012) 16:71–80. doi: 10.1177/1087054710379735 20837978

[23] Ziereis S Jansen P . Effects of physical activity on executive function and motor performance in children with adhd. Res Dev Disabil. (2015) 38:181–91. doi: 10.1016/j.ridd.2014.12.005 25561359

[24] Ji H Wu S Won J Weng S Lee S Seo S . The effects of exergaming on attention in children with attention deficit/hyperactivity disorder: randomized controlled trial. JMIR Serious Games. (2023) 11:e40438. doi: 10.2196/40438 37159253 PMC10206614

[25] Kadri A Slimani M Bragazzi NL Tod D Azaiez F . Effect of taekwondo practice on cognitive function in adolescents with attention deficit hyperactivity disorder. Int J Environ Res Public Health. (2019) 16:204. doi: 10.3390/ijerph16020204 30642062 PMC6352161

[26] Memarmoghaddam M Torbati H Sohrabi M Mashhadi A Kashi A . Effects of a selected exercise programon executive function of children with attention deficit hyperactivity disorder. J Med Life. (2016) 9:373. doi: 10.17918/00002792 27928441 PMC5141397

[27] Hoza B Smith AL Shoulberg EK Linnea KS Dorsch TE Blazo JA . A randomized trial examining the effects of aerobic physical activity on attention-deficit/hyperactivity disorder symptoms in young children. J Abnormal Child Psychol. (2015) 43:655–67. doi: 10.1007/s10802-014-9929-y PMC4826563 25201345

[28] Bustamante EE Davis CL Frazier SL Rusch D Fogg LF Atkins MS . Randomized controlled trial of exercise for adhd and disruptive behavior disorders. Med Sci sports Exercise. (2016) 48:1397. doi: 10.1249/MSS.0000000000000891 PMC4911251 26829000

[29] Choi JW Han DH Kang KD Jung HY Renshaw PF . Aerobic exercise and attention deficit hyperactivity disorder: brain research. Med Sci sports Exercise. (2015) 47:33. doi: 10.1249/MSS.0000000000000373 PMC5504911 24824770

[30] Chou C-C Huang C-J . Effects of an 8-week yoga program on sustained attention and discrimination function in children with attention deficit hyperactivity disorder. PeerJ. (2017) 5:e2883. doi: 10.7717/peerj.2883 28097075 PMC5237364

[31] Benzing V Schmidt M . The effect of exergaming on executive functions in children with adhd: A randomized clinical trial. Scandinavian J Med Sci sports. (2019) 29:1243–53. doi: 10.1111/sms.13446 31050851

[32] Liang X Qiu H Wang P Sit CH . The impacts of a combined exercise on executive function in children with adhd: A randomized controlled trial. Scandinavian J Med Sci sports. (2022) 32:1297–312. doi: 10.1111/sms.14192 35611615

[33] Jensen PS Kenny DT . The effects of yoga on the attention and behavior of boys with attention-deficit/hyperactivity disorder (Adhd). J attention Disord. (2004) 7:205–16. doi: 10.1177/108705470400700403 15487477

[34] Nejati V Derakhshan Z . The effect of physical activity with and without cognitive demand on the improvement of executive functions and behavioral symptoms in children with adhd. Expert Rev Neurother. (2021) 21:607–14. doi: 10.1080/14737175.2021.1912600 33849353

[35] Pan C-Y Chu C-H Tsai C-L Lo S-Y Cheng Y-W Liu Y-J . A racket-sport intervention improves behavioral and cognitive performance in children with attention-deficit/hyperactivity disorder. Res Dev Disabil. (2016) 57:1–10. doi: 10.1016/j.ridd.2016.06.009 27344348

[36] Silva LAD Doyenart R Henrique Salvan P Rodrigues W Felipe Lopes J Gomes K . Swimming training improves mental health parameters, cognition and motor coordination in children with attention deficit hyperactivity disorder. Int J Environ Health Res. (2020) 30:584–92. doi: 10.1080/09603123.2019.1612041 31081373

[37] van den Berg V Saliasi E de Groot RH Chinapaw MJ Singh AS . Improving cognitive performance of 9–12 years old children: just dance? A randomized controlled trial. Front Psychol. (2019) 10:174. doi: 10.3389/fpsyg.2019.00174 30787899 PMC6372522

[38] Hattabi S Bouallegue M Yahya HB Bouden A . Rehabilitation of adhd children by sport intervention: A Tunisian experience réhabilitation des enfants tdah par le sport: une expérience tunisienne. La Tunisie medicale. (2019) 97:874–81. doi: 10.5812/intjssh.118756 31872398

[39] Rezaei M Salarpor Kamarzard T Najafian Razavi M . The effects of neurofeedback, yoga interventions on memory and cognitive activity in children with attention deficit/hyperactivity disorder: A randomized controlled trial. Ann Appl Sport Sci. (2018) 6:17–27. doi: 10.29252/aassjournal.6.4.17

[40] Chang S-H Shie J-J Yu N-Y . Enhancing executive functions and handwriting with a concentrative coordination exercise in children with adhd: A randomized clinical trial. Perceptual Motor Skills. (2022) 129:1014–35. doi: 10.1177/00315125221098324 35507726

[41] Li Y He Y-C Wang Y He J-W Li M-Y Wang W-Q . Effects of qigong vs. Routine physical exercise in school-aged children with attention-deficit hyperactivity disorder: A randomized controlled trial. World J Pediatr. (2025) 10:1–11. doi: 10.1007/s12519-025-00890-x 40064759

[42] Higgins JPT Thompson SG . Quantifying heterogeneity in a meta-analysis. Stat Med. (2002) 21:1539–58. doi: 10.1002/sim.1186 12111919

[43] Balshem H Helfand M Schünemann HJ Oxman AD Kunz R Brozek J . Grade guidelines: 3. Rating the quality of evidence. J Clin Epidemiol. (2011) 64:401–6. doi: 10.1016/j.jclinepi.2010.07.015 21208779

[44] Lin L Chu H Murad MH Hong C Qu Z Cole SR . Empirical comparison of publication bias tests in meta-analysis. J Gen Internal Med. (2018) 33:1260–7. doi: 10.1007/s11606-018-4425-7 PMC6082203 29663281

[45] Huang H Jin Z He C Guo S Zhang Y Quan M . Chronic exercise for core symptoms and executive functions in adhd: A meta-analysis. Pediatrics. (2023) 151:e2022057745. doi: 10.1542/peds.2022-057745 36510746

[46] Vysniauske R Verburgh L Oosterlaan J Molendijk ML . The effects of physical exercise on functional outcomes in the treatment of adhd: A meta-analysis. J attention Disord. (2020) 24:644–54. doi: 10.1177/1087054715627489 26861158

[47] Zhang ZY Shi P Zhang K Li CY Feng XS . The frontal association area: exercise-induced brain plasticity in children and adolescents and implications for cognitive intervention practice. Front Hum Neurosci. (2024) 18:1418803. doi: 10.3389/fnhum.2024.1418803 39301538 PMC11410640

[48] Chen C Nakagawa S . Recent advances in the study of the neurobiological mechanisms behind the effects of physical activity on mood, resilience and emotional disorders. Adv Clin Exp Med. (2023) 32:937–42. doi: 10.17219/acem/171565 37665079

[49] Feng LL Li BW Yong SS Tian ZJ . Effects of exercise intervention on physical and mental health of children and adolescents with attention-deficit/hyperactivity disorder: A systematic review and meta-analysis based on icf-cy. J Sci Sport Exercise. (2024) 8:1–8. doi: 10.1007/s42978-024-00295-8

[50] Di Liegro CM Schiera G Proia P Di Liegro I . Physical activity and brain health. Genes. (2019) 10:720. doi: 10.3390/genes10090720 31533339 PMC6770965

[51] Shaw P De Rossi P Watson B Wharton A Greenstein D Raznahan A . Mapping the development of the basal ganglia in children with attention-deficit/hyperactivity disorder. J Am Acad Child Adolesc Psychiatry. (2014) 53:780–9.e11. doi: 10.1016/j.jaac.2014.05.003 24954827 PMC10461726

[52] Mueller PJ . Exercise training and sympathetic nervous system activity: evidence for physical activity dependent neural plasticity. Clin Exp Pharmacol Physiol. (2007) 34:377–84. doi: 10.1111/j.1440-1681.2007.04590.x 17324153

[53] Daniela M Catalina L Ilie O Paula M Daniel-Andrei I Ioana B . Effects of exercise training on the autonomic nervous system with a focus on anti-inflammatory and antioxidants effects. Antioxidants. (2022) 11:350. doi: 10.3390/antiox11020350 35204231 PMC8868289

[54] Kim J-S . Effects of Taekwondo Exercise on the Psychological Well-Being of School Children and Young Adults. UNSW Sydney (2001).

[55] Adams DB . Brain mechanisms for offense, defense, and submission. Behav Brain Sci. (1979) 2:201–13. doi: 10.1017/S0140525X00061926

[56] Berridge CW Devilbiss DM . Psychostimulants as cognitive enhancers: the prefrontal cortex, catecholamines, and attention-deficit/hyperactivity disorder. Biol Psychiatry. (2011) 69:e101–e11. doi: 10.1016/j.biopsych.2010.06.023 PMC3012746 20875636

[57] Wang M Yang X Yu J Zhu J Kim H-D Cruz A . Effects of physical activity on inhibitory function in children with attention deficit hyperactivity disorder: A systematic review and meta-analysis. Int J Environ Res Public Health. (2023) 20:1032. doi: 10.3390/ijerph20021032 36673793 PMC9859519

[58] Dobbins M Husson H DeCorby K LaRocca RL . School-based physical activity programs for promoting physical activity and fitness in children and adolescents aged 6 to 18. Cochrane Database systematic Rev. (2013) 2:1–10. doi: 10.1002/14651858.CD007651.pub2 PMC7197501 23450577

[59] Gilbert C . Optimal physical performance in athletes: key roles of dopamine in a specific neurotransmitter/hormonal mechanism. Mech Ageing Dev. (1995) 84:83–102. doi: 10.1016/0047-6374(95)01635-X 8788237

[60] Kruk J Kotarska K Aboul-Enein BH . Physical exercise and catecholamines response: benefits and health risk: possible mechanisms. Free Radical Res. (2020) 54:105–25. doi: 10.1080/10715762.2020.1726343 32020819

[61] McMorris T Hale BJ . Is there an acute exercise-induced physiological/biochemical threshold which triggers increased speed of cognitive functioning? A meta-analytic investigation. J Sport Health Sci. (2015) 4:4–13. doi: 10.1016/j.jshs.2014.08.003

[62] Meeusen R Piacentini MF Meirleir KD . Brain microdialysis in exercise research. Sports Med. (2001) 31:965–83. doi: 10.2165/00007256-200131140-00002 11735681

[63] Lin T-W Kuo Y-M . Exercise benefits brain function: the monoamine connection. Brain Sci. (2013) 3:39–53. doi: 10.3390/brainsci3010039 24961306 PMC4061837

[64] Breitbach S Tug S Simon P . Circulating cell-free DNA: an up-coming molecular marker in exercise physiology. Sports Med. (2012) 42:565–86. doi: 10.2165/11631380-000000000-00000 22694348

[65] Tremblay MS Copeland JL Van Helder W . Influence of exercise duration on post-exercise steroid hormone responses in trained males. Eur J Appl Physiol. (2005) 94:505–13. doi: 10.1007/s00421-005-1380-x 15942766

[66] Qiu C Zhai Q Chen S . Effects of practicing closed-vs. Open-skill exercises on executive functions in individuals with attention deficit hyperactivity disorder (Adhd)—a meta-analysis and systematic review. Behav Sci. (2024) 14:499. doi: 10.3390/bs14060499 38920831 PMC11200859

[67] Ye Y Ning K Wan B Shangguan C . The effects of the exercise intervention on fundamental movement skills in children with attention deficit hyperactivity disorder and/or autism spectrum disorder: A meta-analysis. Sustainability. (2023) 15:5206. doi: 10.3390/su15065206

[68] Liang X Li R Wong SH Sum RK Sit CH . The impact of exercise interventions concerning executive functions of children and adolescents with attention-deficit/hyperactive disorder: A systematic review and meta-analysis. Int J Behav Nutr Phys Activity. (2021) 18:68. doi: 10.1186/s12966-021-01135-6 PMC8141166 34022908

[69] Jiang K Xu Y Li Y Li L Yang M Xue P . How aerobic exercise improves executive function in adhd children: A resting-state fmri study. Int J Dev Neurosci. (2022) 82:295–302. doi: 10.1002/jdn.10177 35274372

[70] Cerrillo-Urbina AJ García-Hermoso A Sánchez-López M Pardo-Guijarro MJ Santos Gómez J Martínez-Vizcaíno V . The effects of physical exercise in children with attention deficit hyperactivity disorder: A systematic review and meta-analysis of randomized control trials. Child: care Health Dev. (2015) 41:779–88. doi: 10.1111/cch.12255 25988743

[71] Berwid OG Halperin JM . Emerging support for a role of exercise in attention-deficit/hyperactivity disorder intervention planning. Curr Psychiatry Rep. (2012) 14:543–51. doi: 10.1007/s11920-012-0297-4 PMC3724411 22895892

[72] Chen J-W Du W-Q Zhu K . Optimal exercise intensity for improving executive function in patients with attention deficit hyperactivity disorder: systematic review and network meta-analysis. Eur Child Adolesc Psychiatry. (2024) 26:1–22. doi: 10.1007/s00787-024-02507-6 38922348

[73] Tsai Y-J Hsieh S-S Huang C-J Hung T-M . Dose-response effects of acute aerobic exercise intensity on inhibitory control in children with attention deficit/hyperactivity disorder. Front Hum Neurosci. (2021) 15:617596. doi: 10.3389/fnhum.2021.617596 34220467 PMC8249764

[74] Medicine ACoS . Acsm’s Guidelines for Exercise Testing and Prescription. Lippincott williams & wilkins (2013). 10.1249/JSR.0b013e31829a68cf 23851406

